# Damage and Failure Analysis of Polymer-Based Composites

**DOI:** 10.3390/polym17030272

**Published:** 2025-01-22

**Authors:** Xiaoquan Cheng, Wenjun Huang, Qian Zhang

**Affiliations:** 1 School of Aeronautic Science and Engineering, Beihang University, Beijing 100191, China; 2 AVIC China Helicopter Design and Research Institute, Jingdezhen 333001, China; chrdihwj@163.com; 3 School of Mechanical Engineering, Hefei University of Technology, Hefei 230009, China; zq_hfut@hfut.edu.cn

## 1. Introduction

Polymer-based composites are widely used in aerospace, automotive, construction and wind energy fields due to their high specific strength and stiffness, corrosion resistance, and customizable properties. However, the mechanical behaviors of these materials are complex, and various types of damage, such as delamination, matrix cracking, interface debonding and fiber breakage, can occur. These types of damage can significantly affect the mechanical performance and long-term durability of the structure. Therefore, in-depth research on the damage and failure mechanisms of polymer-based composites is of great significance for improving structural efficiency, reducing total life-cycle costs and extending service life.

In recent times, damage and failure analysis of polymer-based composites has remained a key research topic. Researchers focus not only on the influence of the material’s microstructure on the damage mechanism [1], but also on developing new numerical methods, damage evolution models and nondestructive testing technologies to achieve more precise failure predictions and performance improvement [2,3]. For instance, mesoscopic and multi-scale models have been widely applied to analyze the damage evolution process of composites under different loading conditions [4]. And experimental methods based on technologies such as Digital Image Correlation (DIC) and X-ray Computed Tomography (CT) imaging have also become effective means for studying the internal damage evolution of composite materials [5]. Furthermore, with the rapid development of artificial intelligence and machine learning technologies, intelligent damage monitoring, analysis and prediction techniques for composite are beginning to show great potential [6].

Based on the research at material level, further investigation into the damage and failure of composite structures is also necessary. Researchers usually focus on typical composite structures, such as sandwich structures, stiffened panels, joints and repaired structures, addressing issues including failure mechanisms, damage tolerance performance, numerical models and design methods [7]. The failure of polymer composite structures is also closely related to loading conditions, external environmental factors, and the manufacturing processes of the composites. Therefore, many researchers are focused on the influence of environmental conditions, such as temperature, humidity, and chemical agents, on the performance of composites, aiming to further improve damage prediction models and design methods [8,9]. Additionally, many studies are dedicated to improving manufacturing processes or developing new material systems, such as novel polymer matrixes, hybrid fibers, natural fiber reinforcements or gradient functional materials to enhance the performance of composite structures, reduce production costs or minimize environmental impact [10,11,12].

Twenty manuscripts were submitted for this Special Issue and twelve research papers were finally accepted. This Special Issue mainly focuses on the damage and failure mechanisms of different polymer-based composites and their influence factors, while also discussing novel damage assessment methods and numerical models based on various advanced testing technologies. We aim to provide researchers with the latest findings and offer theoretical or practical guidance for the failure analysis and design of composite materials and structures.

## 2. An Overview of Published Articles

Four articles covered the topics of damage and failure mechanism of polymer-based composites:

Zheng et al. (contribution 1) conducted an investigation into the sand erosion resistance of polyurethane films applied to helicopter rotor blades. Their findings indicated that the impact angle played a crucial role in determining the erosion patterns and damage mechanisms. At lower angles, sand cutting dominated, causing significant surface deformation and eventual shedding of larger polyurethane fragments. However, higher impact angles resulted in concentrated impact damage with smaller debris being shed. Additionally, the study highlighted that volume loss, as a direct measurement approach, provided a more reliable evaluation of sand erosion resistance by eliminating inaccuracies caused by embedded sand particles. The researchers also developed a predictive equation to model volume loss based on experimental observations, offering a robust tool for forecasting erosion behavior.

An et al. (contribution 2) investigated the tensile and compressive properties of woven fabric CFRP laminates containing three-dimensional microvascular channels, which were designed for self-healing applications. The study found that the laminates tend to fail at the location where the thickness-direction micro channels are arranged. While the micro channels minimally affect the overall stiffness, they do lead to a reduction in the strength of the laminate. Several FE models were developed to simulate the mechanical behavior of CFRP laminates containing micro-channels. It was found that resin-rich regions around the microvascular channels, particularly in the warp and weft yarns of the woven fabric composite layers, were identified as a key consideration when establishing FE models. These regions must be accurately represented in the models to predict the mechanical properties of the laminates correctly. Using the validated FE model, the parameter study showed that the reduction in the strength of the laminates exhibited a roughly linear relationship with the diameter of the microvascular channels in the thickness direction, within a typical design range of 0.1 to 1 mm.

Premanand et al. (contribution 3) conducted interrupted constant-amplitude three-point bending fatigue experiments on carbon-fiber satin-fabric-reinforced poly-ether-ketone-ketone (CF-PEKK) composites, using an ultrasonic fatigue testing (UFT) system operating at a cyclic frequency of 20 kHz. The study measured parameters such as the specimen’s surface temperature, acoustic activity and ultrasonic generator resonance during cyclic loading. Additionally, stiffness measurements and volumetric damage characterization using 3D X-ray microscopy (XRM) were carried out to trace damage initiation and accumulation. The results showed that UFT significantly shortens the testing duration compared to conventional fatigue tests, proving advantageous for high-cycle fatigue regimes. Damage progression commenced at the surface and progressively spread towards the interior of the specimen, with surface temperature and acoustic activity serving as critical indicators of damage progression. And it was important to consider thermal influences on the material during testing, as variations in temperature can significantly affect the mechanical properties and fatigue life of the composite.

Chen et al. (contribution 4) investigated the mechanical behaviors of Zylon fiber-reinforced polymers (ZFRPs) under the influence pulsed magnets. The study involved mechanical testing of ZFRP laminates and the development of a constitutive model that accounted for plastic deformation and progressive damage under magnetic field stresses. This model was applied to investigate the failure of a 95-T double-coil prototype, with results indicating a significant reduction of approximately 45% in both the radial and axial stiffness of ZFRP composites, particularly in the inner layers. The damage incurred by the end ZFRPs was the primary cause of the failure, which aligned closely with the experimental results. Additional magnet systems of different magnetic fields were also analyzed to explore the performance of ZFRP. The article concluded that ZFRP composites were highly susceptible to damage and stiffness reduction under extreme magnetic field conditions. The end ZFRPs of the magnet were particularly vulnerable, and the failure mechanism was closely tied to axial Lorentz forces and transverse strength of the material.

Three articles were related to performances and failure analysis of composite structures:

Zhang et al. (contribution 5) investigated the energy-absorption characteristics of thin-walled polymer composite C-channels used in civil aircraft structures under low-speed axial compression. An experimental study was conducted on specimens with layups of [0/90]_3s_, [45/-45]_3s_, and [45/90/-45/0]_3_, and the thin-walled composite C-channels underwent a stable progressive crushing process, with failure modes including local buckling, fiber breakage, matrix cracks, delamination and corner cracking. Then, stacked shell models were developed after conducting parametric studies on simple single-element models and the numerical results on energy absorption showed less than 6% difference with the test results. It can be seen that thin-walled polymer composite C-channels offered excellent energy-absorbing characteristics, making them suitable for use in energy dissipation applications such as crashworthy structures.

Yu et al. (contribution 6) conducted experiments to study the performance of composite helicopter rotor blades under different ballistic impact conditions. The test results showed that failures were particularly prevalent at the bullet incidence and exit positions of the blade. At the incidence position, the damage was mainly local shear fracture, while at the exit position, fiber tensile fracture was the dominant failure mode. And ballistic damage size decreased as the incident angle of the projectile increased. A numerical model was also established and the verified model was used to conduct the parameter study. It was found that the structural stiffness near the exit position of the blade had a significant impact on the size of the damage. Blades with higher stiffness near the exit experienced larger damage sizes at the exit point. The numerical model provided a reliable tool for predicting ballistic damage and assessing the survivability of composite helicopter blades under attack, which had significant implications for improving the design of composite structures in helicopters.

Shin and Huang (contribution 7) focused on a novel structural health monitoring method for the widely used single-lap composite adhesive joint, doping the adhesive with carbon nanotubes and monitoring the joint’s electrical resistance change. Tensile fatigue tests were conducted and electrical resistance of the joints were measured. In addition, the debonding area was recorded using a liquid penetrant technique and the fracture surfaces were examined by scanning electron microscopy (SEM). The results showed that the debonding area showed a linear relationship with the fatigue life expended. However, the relationship between resistance change and debonding was not straightforward, as it followed two different trends, which were associated with different failure mechanisms. Therefore, the percentage voltage change was not a valid quantifying parameter for the fatigue life of a single-lap composite adhesive joint.

Two articles studied the failure of polymer composites under different environments:

Yan et al. (contribution 8) utilized fiber Bragg grating (FBG) sensors to monitor the damage progression of composites, which were embedded in CFRP laminates and can provide real-time data about the deformation and failure progression during the tests. In high-temperature tensile tests, the FBG sensors successfully monitored the damage evolution, including matrix cracking, fiber breakage, and delamination even though the tensile strength of the CFRP composites decreased by 75%. The tests showed the high reliability of FBG sensors in tracking damage under complex conditions and potential of using FBG sensors for long-term structural health monitoring in, for example, aviation insulation layers. In addition, finite element models were established to study the thermal decomposition behavior of the composite materials at high temperatures.

Fan et al. (contribution 9) conducted hydrothermal accelerate aging tests and natural storage tests to study the influence of hydrothermal environments on FRP composites. The results of various testing techniques showed that the plasticization of the resin and interface weakening were major contributors to the decrease in shear strength. Then, a storage life prediction model was established to convert the hydrothermal accelerated aging time to natural storage life. Based on the failure criterion of a specific reduction in shear strength, the storage life of composite can be predicted. This research provided a reliable approach for predicting the storage life of carbon fiber/epoxy composites under real-world conditions. And it was particularly valuable for aerospace applications, whose long-term durability and structural integrity were very critical.

Three articles contribute to the improvement of manufacturing processes or the development of new materials:

Wang et al. (contribution 10) employed three different epoxy resin systems to study the synergistic effects of liquid rubber (CTBN) and thermoplastic particles (PEK-C) for toughening epoxy resin (EP). The structure, curing behavior, thermal properties, impact and bending performances of these EP systems were systematically investigated. The experimental results demonstrated that the combination of PEK-C and CTBN in the epoxy resin led to superior toughness compared to when these agents were used individually, as PEK-C contributed to improved thermal stability while CTBN significantly enhanced the fracture toughness and impact resistance of the resin. Specifically, the synergistic toughening system exhibited improved mechanical properties (including increased tensile strength, elongation at break, and impact strength), enhanced thermal stability (the resin maintained good performance under elevated temperatures, with better resistance to thermal degradation) and better phase morphology (the combination of these toughening agents led to a more uniform distribution of the particles within the resin matrix, which contributed to its improved overall toughness).

Xiao et al. (contribution 11) prepared itaconic acid epoxy resin (EIA) blended with bisphenol A diglycidyl ether (DGEBA) at various ratios, creating bio-sourced composites with different degrees of greenness. Compression, bending and interlaminar shear strength properties of these composites were tested after 48 h of aging at 80 °C under water immersion. The test results showed that the T700/EIA-30 (30% of EIA epoxy) composites showed better dimensional stability and strength retention after hydrothermal aging than traditional petroleum-based composites. The findings highlighted the potential of bio-based epoxy resins to replace petroleum-based counterparts, offering a greener and more sustainable solution with higher performance for future composite material development.

Lee and Yoo (contribution 12) studied the mechanical strength of liquid silicone rubber (LSR) with added carbon black (CB) particles and their resistance to degradation when exposed to oils. The test results showed that the mechanical properties of the LSR/CB composite, including tensile strength and modulus, improved as the CB content increased. When exposed to oil, the swelling rate and oil absorption were significantly reduced in the LSR/CB composites. And when subjected to high-temperature conditions, the deterioration rate in terms of both mechanical strength and oil absorption was also reduced compared to neat LSR. The improved properties of CB/LSR composites were mainly due to the improvement of the crosslink density and structure of the polymer matrix by CB, making them suitable for various industrial applications, and they are also expected to perform well in oil-exposed environments.

## 3. Conclusions

A thorough understanding of the damage and failure mechanisms of composites is fundamental to improving the performance of composite structures and achieving optimized designs. In this Special Issue, we present the latest research findings on damage and failure analysis of polymer-based composites, covering a wide range of important topics from the damage mechanisms of composite materials and structures to the enhancement of durability in high-performance materials, utilizing various advanced analytical methods and testing technologies. These studies are derived from practical engineering applications or reflect engineering demands. And the results indicate that through advanced analysis, design or monitoring technologies, the performances of composites are expected to be significantly improved in many aspects, demonstrating their broad potential in applications. These studies provide a technical basis or practical guidance for better design and application of related composite materials or structures.

In the future, we look forward to more studies on nanotechnology, smart materials, intelligent monitoring and analysis technology, bio-derived or eco-friendly materials and other fields. Combined with the evolving damage and failure analysis methods, polymer-based composite materials can play a greater role in more applications and make more contributions to global engineering design and sustainable development.

## List of Contributions

1. Zheng, L.; Fan, J.; Gong, Q.; Sun, W.; Jia, X. Sand Erosion Resistance and Failure Mechanism of Polyurethane Film on Helicopter Rotor Blades. *Polymers* **2023**, *15*, 4386. https://doi.org/10.3390/polym15224386.
2. An, Z.; Cheng, X.; Zhao, D.; Ma, Y.; Guo, X.; Cheng, Y. Tensile and Compressive Properties of Woven Fabric Carbon Fiber-Reinforced Polymer Laminates Containing Three-Dimensional Microvascular Channels. *Polymers* **2024**, *16*, 665. https://doi.org/10.3390/polym16050665.
3. Premanand, A.; Prescher, M.; Rienks, M.; Kirste, L.; Balle, F. Online and Ex Situ Damage Characterization Techniques for Fiber-Reinforced Composites under Ultrasonic Cyclic Three-Point Bending. *Polymers* **2024**, *16*, 803. https://doi.org/10.3390/polym16060803.
4. Chen, S.; Peng, T.; Han, X.; Cao, Q.; Xiao, H.; Li, L. Mechanical Behaviors of Polymer-Based Composite Reinforcements within High-Field Pulsed Magnets. *Polymers* **2024**, *16*, 722. https://doi.org/10.3390/polym16050722.
5. Zhang, X.; Mou, H.; Song, S.; Feng, Z. An Investigation of the Energy-Absorption Characteristics of Thin-Walled Polymer Composite C-Channels: Experiment and Stacked Shell Simulation. *Polymers* **2024**, *16*, 2099. https://doi.org/10.3390/polym16152099.
6. Yu, G.; Li, X.; Huang, W. Performance and Damage Study of Composite Rotor Blades under Impact. *Polymers* **2024**, *16*, 623. https://doi.org/10.3390/polym16050623.
7. Shin, C.-S.; Huang, S.-H. Evaluation of Fatigue Damage Monitoring of Single-Lap Composite Adhesive Joint Using Conductivity. *Polymers* **2024**, *16*, 2374. https://doi.org/10.3390/polym16162374.
8. Yan, G.; Wan, B.; Huang, H.; Li, W. Damage and Failure Monitoring of Aerospace Insulation Layers Based on Embedded Fiber Bragg Grating Sensors. *Polymers* **2024**, *16*, 3543. https://doi.org/10.3390/polym16243543.
9. Fan, J.; Zhang, Q.; Chen, X.; He, Y. Interlaminar Shear Strength Change and Storage Life Prediction of Carbon Fiber/Epoxy Composites with Hygrothermal Accelerated Aging. *Polymers* **2024**, *16*, 1109. https://doi.org/10.3390/polym16081109.
10. Wang, Z.; Lai, Y.; Xu, P.; Ma, J.; Xu, Y.; Yang, X. Synergistic Effects of Liquid Rubber and Thermoplastic Particles for Toughening Epoxy Resin. *Polymers* **2024**, *16*, 2775. https://doi.org/10.3390/polym16192775.
11. Xiao, K.; Fang, Y.; Wang, Z.; Ni, N.; Liu, Z.; Kim, S.; An, Z.; Lyu, Z.; Xu, Y.; Yang, X. Bio-Sourced, High-Performance Carbon Fiber Reinforced Itaconic Acid-Based Epoxy Composites with High Hygrothermal Stability and Durability. *Polymers* **2024**, *16*, 1649. https://doi.org/10.3390/polym16121649.
12. Lee, B.-J.; Yoo, H.-M. Effects of Carbon Black on Mechanical Properties and Oil Resistance of Liquid Silicone Rubber. *Polymers* **2024**, *16*, 933. https://doi.org/10.3390/polym16070933.

## Data Availability

Not applicable.
